# Case of Varicella

**Published:** 1818-06

**Authors:** 

**Affiliations:** Walmer, Kent


### CASE of VARICELLA.

By Dr. M'Arthur, M.D. F.L.S. Walmer, Kent.

THE frequently alleged failure of Vaccination, in preventing small pox, is a matter of deep regret, because these reports prevent some parents from submitting their children to vaccination, since it does not promise them absolute security; and, therefore, in many parts of the kingdom, partly from this cause, and partly from the want of agreement amongst the medical practitioners themselves, inoculation for small-pox is occasionally practised; and thus a disease continues to be perpetuated which the friends of humanity had hoped would have been banished by this time from the catalogue of human ills.

Within the last nine years my attention has been directed towards the supposed cases of small-pox which had succeeded vaccination, and many instances have been pointed out to me, but the disease in every case proved only a severe degree of chicken-pox; and I should have been led to suppose, that small-pox as rarely occurred in a person that had been properly vaccinated, as after the inoculated small-pox itself, were it not proved to the contrary by the evidence of men of the first talents and respectability.

It is a trite observation, that diseases occurring during the prevalence of any epidemic, partake, in a considerable degree, of its nature and severity; and I have often remarked varicella to be more severe when it has occurred at the time a bad kind of small-pox or measles has prevailed.

The preceding observations are intended to introduce a Case of chicken-pox which for some days had deceived me; and had I quitted my patient on the fourth day of the eruption, I should have left her, persuaded that the disease was a case of legitimate small-pox, although some of the symptoms appeared earlier than common. I subjoin the Case in the ipsissima verba of the memoranda I took at the time.

"I was this day, the 17th of June, 1816, requested to visit Miss M'K——, aged about 13 years. Had been vaccinated ten years ago at the Naval Hospital, and a cicatrice is evident on her right arm. Mr. Howell, a surgeon in the royal navy, and a friend of the family, states, that on Saturday last, the 15th instant, she complained of a loss of appetite, with pain and uneasiness in the region of the stomach; skin hot and dry, head-ach, anxiety, thirst, and some difficulty in swallowing, but unattended with pain. On Sunday the 16th, the febrile symptoms had increased; there was much delirium; pulse 150. Mr. H. abstracted ten ounces of blood from the arm, and opened her bowels with the sulphate of magnesia. This day delirium was less; heat of skin abated; pulse 150. An erythematous inflammation occupies the face, neck, arms, and particularly the abdomen; but has not extended to the lower extremities. To be kept cool, and her bowels to be opened with the sulphate of magnesia. Tuesday the 18th, a crop of small pustules appears on the face and neck, and a few are appearing on the arms; the inflammation extends to the lower extremities; delirium gone. Wednesday the 19th, the eruption on the face and upper extremities very full; a considerable number has appeared on the lower extremities; no pustules on any part of the abdomen; the efflorescence has nearly disappeared on the face and arms; on the abdomen it is of a less vivid colour, and very much resembles the red-gum in children; pulse 100; tongue clean; some enlargement of the tonsils, and pain in swallowing. Thursday the 20th, pustules every where increasing in size, and more elevated; eye-lids swollen; soreness of the throat, and increased flow of saliva; pulse 96. Friday the 21st, pustules still increasing, and filling with a straw-coloured fluid; confluent on each side of the nose; eyes closed; pulse 100. Saturday the 22d, pustules on the face depressed and beginning to blacken; on the extremities they are stationary, containing a whey-like fluid; face less tumid; soreness of the throat abated; can open her eyes, and seems altogether better. Sunday the 23d, pustules on the face dry and shrivelled, those on the arms depressed; on the lower extremities they continue much as before: neither ptyalism nor soreness of the throat. Monday, the scabs on the face are already nearly dropped off, leaving a horny elevation occupying the centre of each pustule; the eruption on the extremities disappearing.—In a few days after this period, the scabs had entirely dropped off, leaving her in perfect health. The erythematous inflammation which preceded the eruption of the pustules disappeared, as the latter increased in size. It is singular, the pustules did not extend to any part of the abdomen."

Walmer, Deal, March 30, 1818.

